# Cone-beam computed tomography investigation of middle mesial canals and isthmuses in mandibular first molars in a Chinese population

**DOI:** 10.1186/s12903-020-01126-2

**Published:** 2020-05-08

**Authors:** Shuaimei Xu, Junfeng Dao, Zhongjun Liu, Zhiyong Zhang, Yu Lu, Xiongqun Zeng

**Affiliations:** 1 Department of Endodontics, Stomatological Hospital, Southern Medical University, No 366 Jiangnan Avenue South, Guangzhou, 510280 Guangdong China; 2 Department of Prosthodontics, Stomatological Hospital, Southern Medical University, Guangzhou, China; 3 Department of Oral and Maxillofacial Radiology, Stomatological Hospital, Southern Medical University, Guangzhou, China

**Keywords:** Middle mesial canal, Isthmus, Mandibular molar, Cone-beam computed tomography

## Abstract

**Background:**

While there is ample research into the anatomy of mandibular molars, little is known regarding isthmuses and middle mesial (MM) canals in Chinese populations. The goal of this study was to determine the prevalence of MM canals and isthmuses in the mesial root of mandibular first molars using Cone-beam Computed Tomography.

**Methods:**

Cone-beam Computed Tomography images of 357 mature mandibular first molars were retrospectively analyzed. Presence of isthmuses and MM canals, and the length of isthmuses in the mesial root were recorded. Meanwhile, we also recorded possible correlated factors such as demographics, side of mandible, presence of separated distal-lingual roots.

**Results:**

Of these 357 teeth, 209 showed evidence of either complete or partial communication in the mesial root. Of these, 11(3.1%) exhibited true MM canals while 198(55.5%) exhibited isthmuses. Sex or side of mandible was not correlated with the prevalence of isthmuses (*P* > 0.05). However, there was a significant association between the presence of a distal-lingual root and the prevalence of such communication (*P* < 0.001). The average length of isthmuses was 4.3 ± 3.1 mm.

**Conclusions:**

We detected high rate of isthmuses and low rate of MM canals in mesial roots of mandibular first molars, which is important as such areas should be identified and cleaned during root canal treatment.

## Background

Disinfecting the root canal system is essential during endodontic treatment to ensure optimal patient outcomes, with particular efforts made to remove any infected pulp or debris from areas of operation. Owing to the presence of canals and communications between canals, however, thoroughly sterilizing this environment is often impossible [1]. As the positioning and number of such canals vary on a patient-by-patient basis, this further increases the complexity for clinicians aiming to achieve safe and sterile operation.

An isthmus is identified as a narrow, ribbon-shaped communication between two root canals that contains pulp tissue. Isthmuses in the root canal system, specifically of the maxillary and mandibular molars, may contain necrotic debris, tissue remnants, or organic substrates that facilitate the growth of microorganisms. Rates of treatment success have been found to be lower for teeth with a prepared isthmus than for teeth without any isthmus, and as such, there is a key need to explore isthmus anatomy for each tooth to better understand and control surgical outcomes and procedures in patients [2].

Many researchers to date have investigated the complexities of mesial root anatomy in mandibular molar teeth [3–5]. The MM canal varies in morphology and anatomic location and its prevalence is affected by ethnicity, age group and the different investigative methods used in the studies [6]. As such, the prevalence of isthmuses or the middle mesial (MM) canals in the mesial root of these teeth still remain controversial.

Indeed, many approaches have been employed for assessing the presence of MM canals, with estimated frequencies from 0.26 to 53.8% [6–9]. Isthmus frequencies have been found to range from 20 to 70% [2, 10–12]. These extensive ranges may result from individual study variations in investigative methods, patient age, or patient ethnicity. However, little is specifically known regarding isthmuses and middle mesial (MM) canals in Chinese populations. As such, this study had 3 objectives:

1. Using cone-beam computed tomographic (CBCT) images to determine the prevalence of MM canals and isthmuses in the mesial root of mandibular first molar.
2. To correlate these prevalence rates with key demographic variables, and with mandible side as well as with separated distal-lingual root presence.
3. To assess the isthmus length and the type of MM canals in the mesial root of mandibular first molar.

## Methods

### Study design, source and period

A retrospective cross-sectional study design was employed from January 1, 2016, to June 1, 2017 in the database of the oral and maxillofacial radiology department of Stomatological Hospital, Southern Medical University.

### Sample size and sampling procedures

Sample calculation was based on single sample rate calculation formula: $\mathrm{n}={\left(\frac{Z_{\propto }}{\delta}\right)}^2\pi \left(1-\pi \right)=325$ where overall prevalence *π* = 69.6%, α = 0.05, δ = 0.05, one-tailed, where *π* is from previous studies [7, 11] using 95% confidence intervals. In order to get high precision in this study, 10% of the samples should be added on the basis of sample size calculated by the above formula as the final sample size. Therefore, a total of 357 CBCT images of mandibular first molars from 334 native Chinese patients (Ages 9–81) were selected.

### Inclusion criteria and exclusion criteria

Included criteria were: CBCT images of mandibular first molars; age is 9–81 years old; exclude criteria were: teeth with any crown restorations, root canal therapy, severe calcification in the root canal systems, internal root resorption and cases with distorted image. The requirements for using CBCT was due to insufficient information from conventional radiographs for diagnosis and treatment planning. CBCT scans were mainly used for surgical removal of impacted teeth, implant surgery or orthodontic treatment. Therefore, the subjects included in this study did not receive unnecessary radiation for root canal anatomy evaluation. All chosen teeth exhibited complete root formation and closed apices.

### Variables

Sex, age, whether exists separated distal-lingual root cana, side of mandible, MM canals and isthmuses in the mesial root of mandibular first molar were recorded. Patients were divided into 4 groups based on age: group A (< 20 years), group B (20–39 years), group C (40–59 years), and group D (≥60 years).

### Radiographic image acquisition and analysis

**Fig. 1:**
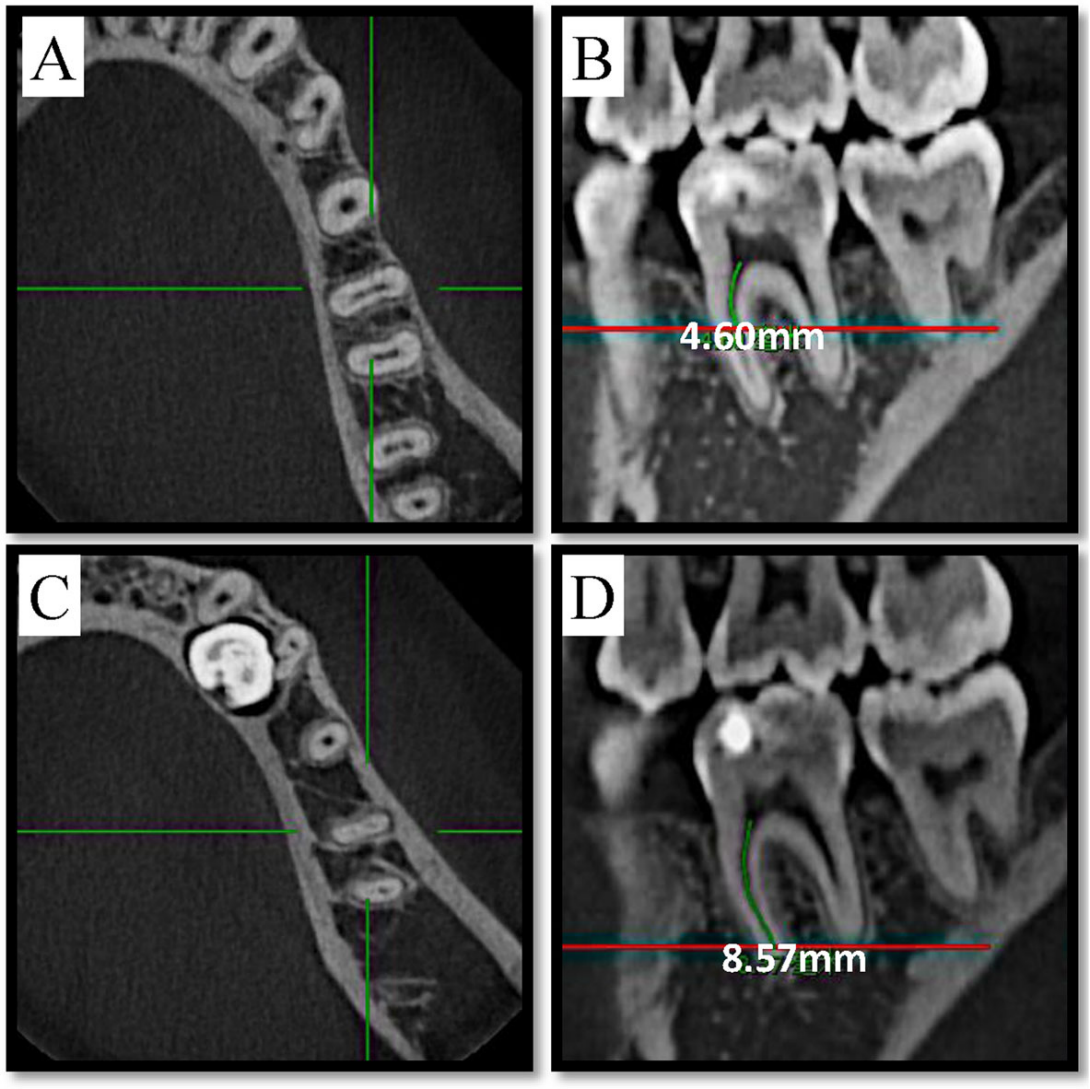
Measurement of isthmus length using Carestream Software’s built-in measuring tool. The red line in figure (**b**) corresponds to the level of figure (**a**), the red line in figure (**d**) corresponds to the level of figure (**c**). (**a**) The first cross-sectional picture exhibiting the isthmus in coronal-apical direction, which corresponds to the upper edge of isthmus. **b** linear distance of the upper edge of isthmus to the root canal orifice is 4.60 mm. **c** The first cross-sectional picture where the isthmus disappears in coronal-apical direction, which corresponds to the lower edge of isthmus. **d** linear distance of the lower edge of the isthmus to the root canal orifice is 8.57 mm. Thus length of the isthmus = linear distance of the lower edge of the isthmus to the root canal orifice - linear distance of the upper edge of the isthmus to the root canal orifice, which means 3.97 mm = 8.57 mm - 4.60 mm

A NewTom VGI (QR Srl, Verona, Italy) CBCT unit with an isotropic voxel size of 0.125 mm, 110 kV and 2.79 mA as exposure parameters, 8 cm × 8 cm as field of view and 3.6 s as exposure time was used for this study. In a dimly lit room, the Carestream Dental Imaging Software 3D module v2.4 (Carestream Health, Inc., Rochester, NY) was used to inspect CBCT images on a Dell Professional P2213 workstation (Dell, Round Rock, TX) using a 22-in. monitor at a resolution of 1680 × 1050. Two endodontists with five years of clinical experience, who were helped by a radiologist experienced in endodontics, evaluated all CBCT images independently and discussed for any disagreement until consensus was reached. Image evaluation was conducted in five sessions with a time interval of one week between each session. Data regarding demographics, side of mandible, presence of separated distal-lingual roots, presence of isthmuses and MM canals, and the length of isthmuses (the distance between the lower edge and the root canal orifice minus the distance between the upper edge and the root canal orifice, as shown in Fig. 1) in the mesial root were recorded (Additional file 1: Raw data).

**Fig. 2:**
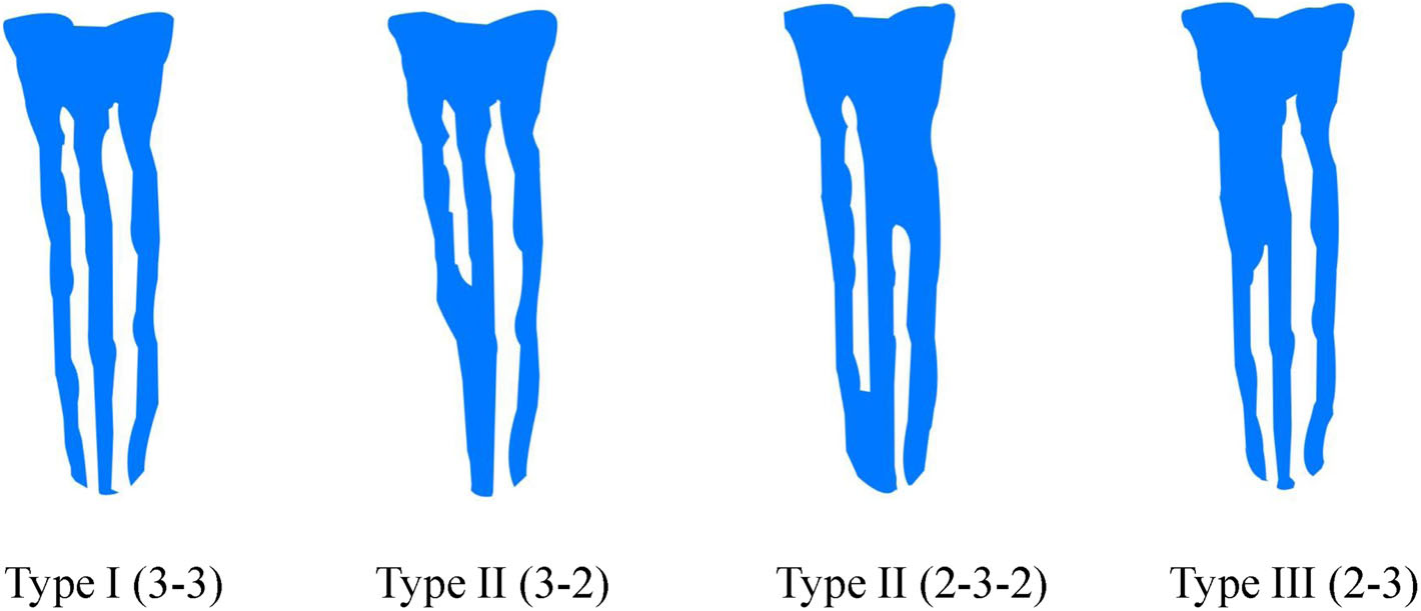
Diagrammatic representation of MM canal types according to the descriptions of Pomeranz

The report of Mehrnaz Tahmasbi [7] was used as a guide for establishing MM canal configurations. A true MM canal was determined to be present if there was a clear, round cross-section visual in the radiographic image located between the MB and ML canals, which was used to identify MM canals without regard for the presence or absence of an isthmus. An isthmus was identified based on the presence of a thin ribbon-shaped communication between the ML and MB canals when inspected in the axial view. The descriptions of Pomeranz [11] were used to identify MM type (Fig. 2). Type I: Independent, three independent root canals were detectable extending to the root apex from the chamber; Type II: Confluent, an MM canal joined to either the mesiolingual or mesiobuccal root canal on its trajectory towards the apex; Type III: Fin, the MM canal orifice was connected to the orifices of the mesiobuccal or mesiolingual canals, but ended in a separate foramen. According to the location of beginning and end, the isthmuses are divided into 7 categories.

### Statistical analysis

All collected data were entered into Microsoft Excel (Microsoft, Redmond, WA), then subsequently analyzed using SPSS v25.0 (SPSS Inc., Chicago, IL). The outputs of descriptive statistical analysis was presented as frequency (percentages) for categorical variables. Chi-squared was used to compare differences in isthmus presence. Unconditional multivariate logistic regression analysis was also run to determine the association between isthmus presence and sociodemographic characteristics. A critical point, *P* < 0.05 at a confidence interval of 95% in the multivariate analysis was used to determine the level of significance.

### Ethics approval

The Institutional Review Board and Ethical Committee of Stomatological Hospital, Southern Medical University approved this study protocol and data acquisition (Date of approval: March 12, 2018. Approval number: 201808).

## Results

**Fig. 3:**
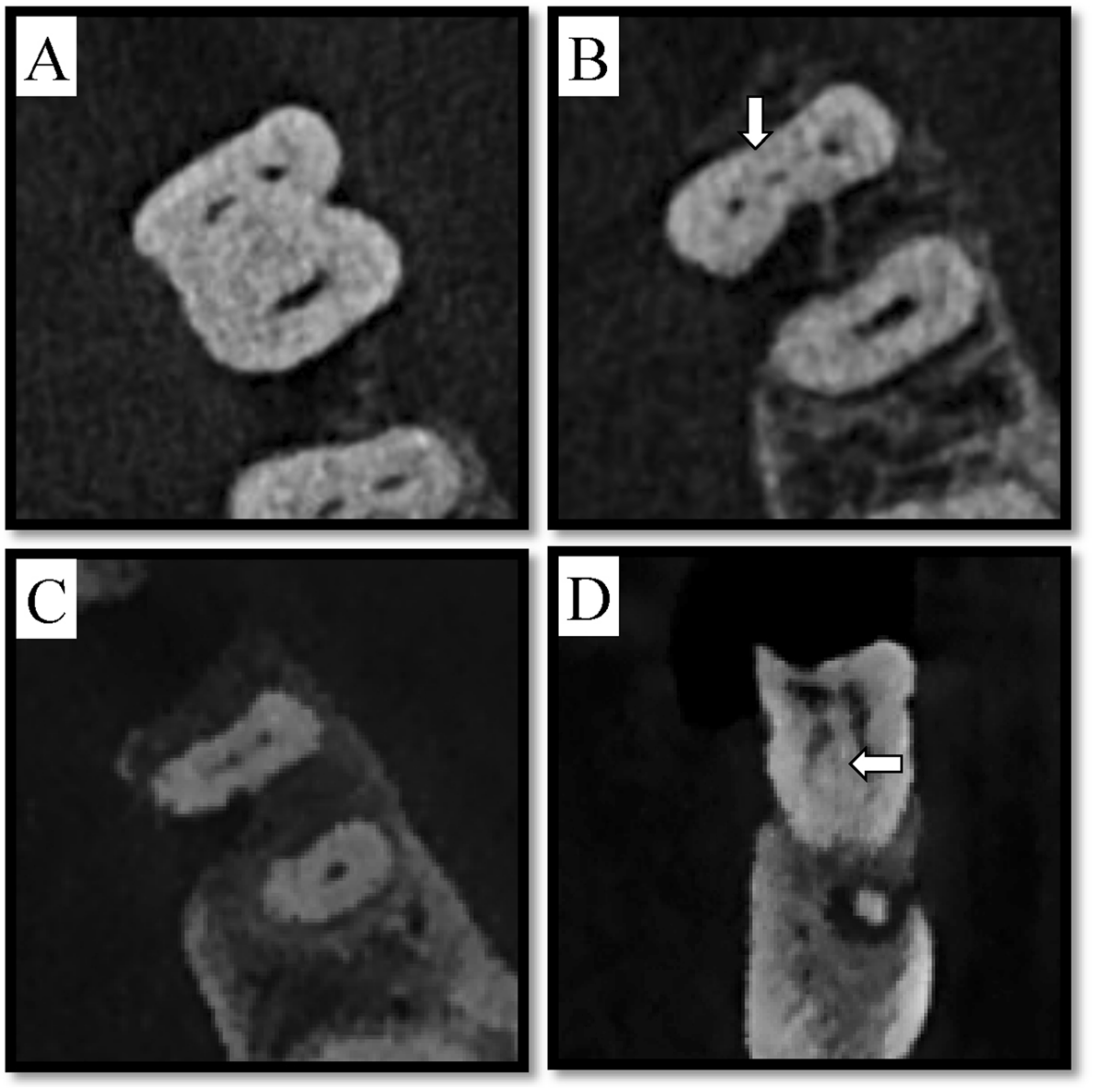
MM canal of tooth #30 at three different axial view levels (cervical, middle, and apical) and in a coronal view image. **a** axial view of cervical third, ) middle third, and **c** apical third. **d** Image of coronal view showing the MM canal. The arrow points to the MM canal

We collected a total of 357 mandibular first molars from 334 patients (mean age = 41.24 years). Of these teeth, 198 (55.5%) had isthmuses, while only 11 (3.1%) had true MM canals (Fig. 3, Table 1). This indicated a total prevalence of isthmus and MM of 58.6%.

**Table 1:** Prevalence of MM and isthmus

	Isthmus	MM	Without communication	Total
Number	198	11	148	357
Prevalence	55.5%	3.1%	41.4%	100%

No significant sex-dependent differences were detected in terms of isthmus prevalence (*P =* 0.839), nor did prevalence differ significantly based on the side of the mandible examined (*P =* 0.282). There was a significantly different prevalence of isthmuses based on age(*P* < 0.001), with patients in group A (< 20 years) and group B (20–39 years) exhibiting higher rates of these conformations than those patients in group C (40–60 years) (*P <* 0.05) and group D (> 60 years) (*P <* 0.001). When compared to group D, the prevalence of isthmuses and MM canals in group C was also significantly higher (*P < 0.001*). When a separated distal-lingual root was present, the prevalence of isthmuses was 41.1%, which was significantly lower than that when such a root was not present (*P* = 0.003) (Table 2).

**Table 2:** Variables associated with isthmus

Independent variables	Isthmus	*P* value
Female(n(%))	101 (54.9)	0.839
Male(n(%))	97 (56.1)	
Age (mean (SD))	37.06 (13.86)	< 0.001
Age (n(%))		< 0.001
< 20 years old	21 (75.0)	
20–39 years	91 (66.4)	
40–59 years	75 (52.4)	
≥ 60 years	11 (22.4)	
Separated distal-lingual root canal(n(%))	30 (41.1)	0.003
No Separated distal-lingual root canal(n(%))	168 (59.2)	
Position right(n(%))	101 (53.2)	0.282
Position left(n(%))	97 (58.1)	

In the unconditional logistics regression model, cases with separated distal-lingual root canal tended to have a higher prevalence of isthmus (*OR =* 0.386, 95%CI (0.215, 0.680)), which is significant in statistics. Besides, age also plays an effect on isthmus prevalence. Comparing to reference group D (≥60 years), isthmus prevalence of group A (*OR =* 13.425, 95%CI (4.439, 46.245)), group B (*OR =* 6.754, 95%CI (3.172, 15.354)) and group C (*OR =* 3.652, 95%CI (1.741, 8.168)) were relatively higher, which indicated that older individuals were significantly less likely to have isthmuses than their younger counterparts. (Table 3).

**Table 3:** Logistics regression result

Variables	*β*	SE	OR(95%CI)	*P* value
(Intercept)	−0.825	0.396	0.438 (0.194, 0.930)	0.038
Female	−0.186	0.235	0.830 (0.523, 1.313)	0.427
Age				
< 20 years old	2.597	0.593	13.425 (4.439, 46.245)	0.000
20–39 years	1.910	0.400	6.754 (3.172, 15.354)	0.000
40–59 years	1.295	0.391	3.652 (1.741, 8.168)	0.001
≥ 60 years	reference			
Position right	−0.084	0.236	0.920 (0.579, 1.461)	0.722
Separated distal-lingual root canal	−0.908	0.294	0.403 (0.225, 0.713)	0.002

**Fig. 4:**
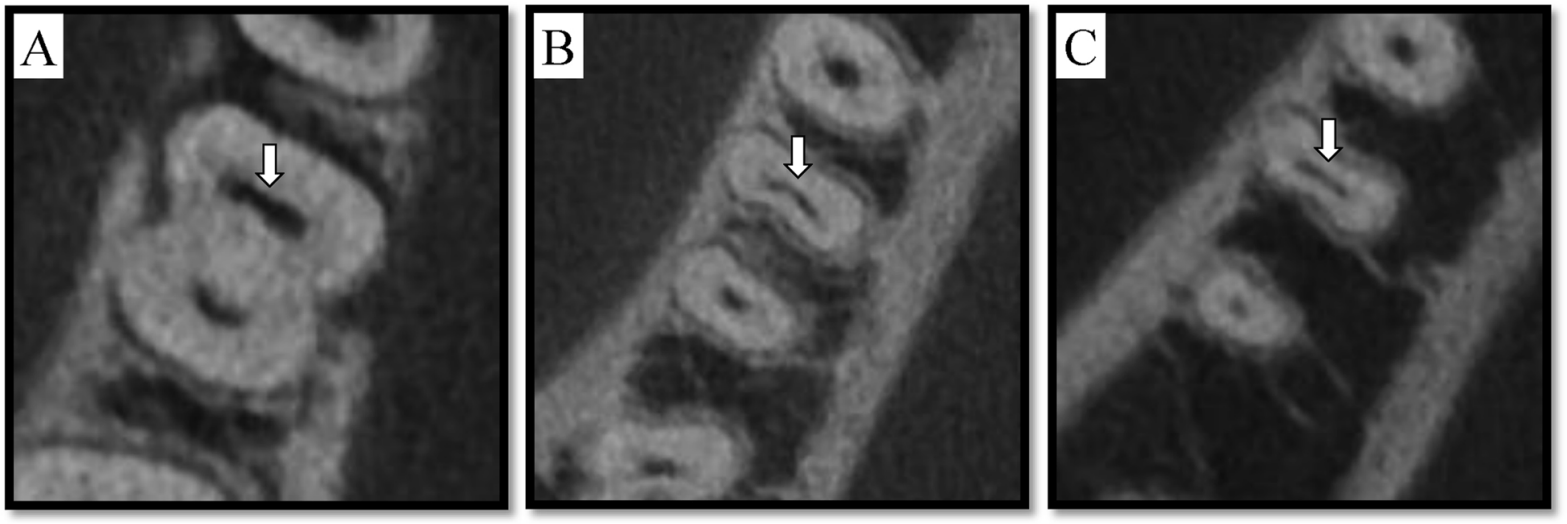
Isthmus of tooth #19 at three different axial view levels (cervical, middle, and apical). **a** axial view of cervical third, **b** middle third, and **c** apical third. The arrow points to the isthmus

Next, the 198 detected isthmus were grouped according to the location along the length of the tooth, allowing us to classify these canals into seven different types that we grouped into three locations for purpose of analysis [7]. Type 1: Cervical third to apical third. Type 2: Cervical third to middle third. Type 3: Cervical third and apical third. Type 4: Confined to cervical third. Type 5: Middle third to apical third. Type 6: Confined to apical third. Type 7: Confined to middle third. The prevalence and length of isthmus were then measured (Table 4). In 47.9% of cases, isthmuses were present in the cervical third, while in 18.5% of cases they were present in the middle third, and in 17.4% of cases, they were present in the apical third (Table 4). In 28 of these cases, complete communication between mesial-buccal canal and mesial-lingual canal was detectable (Fig. 4). The average isthmus length in the mesial root was 4.3 ± 3.1 mm.

**Table 4:** Number (n) and percentage (%) of the isthmus (Type1–7) in different locations and its length (mm)

	Type1	Type2	Type3	Type4	Type5	Type6	Type7	Total
Cervical third	**+**	**+**	**+**	**+**	**–**	**–**	**–**	
Middle third	**+**	**+**	**–**	**–**	**+**	**–**	**+**	
Apical third	**+**	**–**	**+**	**–**	**+**	**+**	**–**	
Number	28	24	11	108	10	13	4	198
Percentage of isthmus	7.9%	6.7%	3.1%	30.3%	2.8%	3.6%	1.1%	55.5%
Length	9.9 ± 3.3	5.9 ± 1.2	5.7 ± 1.7	2.4 ± 0.9	6.2 ± 1.9	2.5 ± 1.4	2.1 ± 1.5	4.3 ± 3.1

+, present; −, absent

True MM canal prevalence was only 3.1% in first mandibular molars. Of these 11 true MM canal cases, 3 exhibited separate orifices distinct from the MB and ML canals, while 2 shared the same orifice with either the MB or ML canal, and the remaining 6 cases branched off from either the middle or the apical third of the MB or ML canals (Table 5).

**Table 5:** Type distribution of MM canals based on descriptions of Pomeranz

MM	Number	Prevalence(%)
Type I	3	0.8%
Type II	6	1.7%
Type III	2	0.6%
Total	11	3.1%

## Discussion

We found the prevalence of isthmuses to be roughly consistent with the findings of previous studies [5, 7, 13], although we detected a lower than expected prevalence of MM canals. There may be a few different potential explanations for this finding. For one, MM canals are defined differently in various studies. For example, Mehrnaz Tahmasbi [7] defined MM canals as we did in the present study, identifying them based on the presence of a distinct round cross-sectional area in radiographic image located in-between the MB and ML canals without regard for whether or not an isthmus was present. In other studies, however, MM canals were considered to be present if 3 canals were evident in the mesial roots [5, 9, 13]. While most studies define isthmuses based on the presence of thin ribbon-shaped communications visible in between the MB and ML canals, others such as Hsu and Kim [14] define types of isthmuses, with a type I isthmus being defined as having either 2 or 3 canals with no noticeable communication, types II and III isthmuses containing 2 and 3 canals, respectively, each with clear communication between the main canals, type IV isthmuses having canals extending into the isthmus area, and type V isthmuses having extensive connections throughout the region. By this definition, MM canals would thus be classified as a form of isthmus, thereby potentially throwing off prevalence for these two types of features in certain studies.

We chose to define isthmuses and MM canals based on the criteria outlined in the study of Mehrnaz Tahmasbi, making an effort to distinguish between isthmuses and true MM canals. We classified isthmuses and true MM canals as specific kinds of communication between the MB and ML canals, and once we established prevalence rates of these features, we analyzed them separately.

An additional factor potentially complicating comparisons between studies is that a range of different techniques have been employed to explore the morphology of mandibular molar root canals, and different approaches have their unique advantages and disadvantages. Previously used methods include plastic casts [12], staining and clearing [15], an operating microscope [16], and micro–CT imaging [3, 4, 17]. These different methods have all yielded different prevalence rates with respect to isthmuses and MM canals. Past findings and relative advantages of some of these approaches are detailed in the following paragraphs.

Microscope-based examinations have determined MM canal frequencies to be from 1 to 37.5% [8, 9, 11, 18, 19]. In a study by Sunil Kim [2], a total of 72/106 teeth exhibited isthmuses, with no evidence of such communications in the remaining 34 teeth. In that same study, cumulative 4-year survival rates were 61.5 and 87.4% when isthmuses were present and absent, respectively. In certain clinical studies, including the above research, MM canals were determined to be present based on the detection of 3 canals upon treatment. However, as certain canal conformations such as a Type IV canal can be present without any true MM canal in evidence, this approach is insufficient as a means of accurately determining the rates of MM canal prevalence.

With respect to micro-CT, this approach has much higher resolution than microscopic, CBCT and clearing-based techniques for accurately observing fine anatomic structures such as MM canals in the resultant 2D and 3D images, as such, micro-CT is considered to be the the gold standard for anatomical studies. Indeed, in some studies highly complex configurations of mandibular first molar root canals were detected, defying standardized classification efforts [20]. When patients were separated by age and assessed for isthmus presence, frequencies for individuals aged 20–39 years, 40–59 years, and ≥ 60 years were 50, 41, and 24%, respectively [21]. However, as micro-CT scans are mostly performed in vitro, the samples are mainly from corpses or Institutional Teeth Banks (with registered age and gender from the donator) which collect teeth extracted due to periodontal diseases, surgery, orthodontic treatment, prosthodontics issues from living people. Due to the limited source and size of the sample, the results from such studies may not be construed as being representative of the general population.

Mehrnaz Tahmasbi used CBCT-based imaging and reported that a MM canal prevalence of 26% in the mesial roots of mandibular first molars, while the frequency of isthmuses was 64.7%. Another study employing CBCT imaging detected an MM canal prevalence rate of just 0.35% (5/1435 teeth), while 26/1435 teeth showed evidence of only a single mesial canal (1.81%) [10]. Wang et al. detected the prevalence of MM to be 2.7% (11/410 teeth) in a western Chinese population using CBCT imaging [22] while Pan et al. found it to be 1.9% (4/208 teeth) in a Malaysian Subpopulation [23]. Marceliano-Alves et al. demonstrated a prevalence of MM to be 7.7% (8/104 teeth) in a Brazilian population using micro-CT technique [24]. Marco Aurélio et al. used micro-CT and found the prevalence of of MM was 18.6% (48/ 258 teeth) [5]. The reasons for these different results may be due to different voxel sizes of micro-CT and CBCT machines and ethnicities of populations according to the latest systematic review [6]. CBCT is primarily utilized in pre-treatment clinical contexts, making it an valuable technique for establishing the presence of isthmuses and MM canals in areas of operative interest. Chavda and Garg proved that CBCT’s detection accuracy of MM is not inferior to magnification and troughing in an in vitro study [25]. Blattner et al. [26] and Zheng et al. [27] reported that CBCT scanning is a reliable method for detecting the second mesiobuccal canal of human maxillary first molars. In the current study, the morphology of root canals can be observed in all directions, and the number of the roots and root canals can be clearly seen in the axial section. CBCT scans can be a useful tool for large-scale studies of root canal anatomy without surgical intervention. For these reasons, we selected CBCT imaging for use in the present retrospective analysis.

We found frequencies of isthmuses to be 55.5%, which is consistent with previous studies [8]. Gender and position of left side or right side are not influencing factor. We did, however, identify a significant relationship between the presence of additional distal-lingual roots and the prevalence of isthmuses. Mandibular first molars most typically have a total of 2 roots and 3 canals in the mesial and distal roots [28]. Different populations seem to exhibit different rates of additional distal-lingual roots, with rates of 22.7% in Japanese patients [29], 15% in patients from Hong Kong [30], and 29% in those from China [31]. A lower rate of just 3.64% was detected in a Turkish Cypriot population. We found in our assessment of a Chinese population that the prevalence of additional distal roots was 20.5%. Of the 73 individual analyzed cases in which a separated distal- lingual root was evident, 41.1% of these showed evidence of isthmus. A significantly higher rate was detected in those cases without this separated distal-lingual root. The reason for this significant variation between these two groups remains uncertain and warrants further investigation.

Our findings indicate that younger individuals are significantly more likely to have isthmuses than their older counterparts, with a prevalence of 75% in those age 20 or less. In their micro-CT reconstruction-based study of isthmus anatomy, Gu et al. [21] found that isthmuses were present significantly more often in younger patients (20–39 years old; 50%) relative to older patients (60+ years old, 24%), with an increasing ratio of partial to complete isthmuses increasing with age [21] . This was also true in a study conducted by Ali Nosrat [9], and are consistent with our results in the present study. The reason for these results may be due to less calcification or secondary dentin deposition of the root canal system in the younger population. As the age increases, the calcification or secondary dentin deposition of the root canal system increases, gradually closing the communication between MB and ML [32]. Together these results indicate that clinicians should take additional time to explore and assess the pulp chamber floor area between MB and ML canals to identify any isthmuses which may be present, especially in younger patients.

When assessing types and lengths of isthmuses, we diverged from the Vertucci standard and instead divided roots into three portions: the cervical, middle, and apical sections. As different sections of the isthmus can have different effects and clinical significance, and this stratification can have important clinical implications. In clinical settings, particularly using microscopic enhancement, cervical sections can be readily dealt with using ultrasonic tips. Such isthmuses were evident in 47.89% of cases, suggesting that isthmuses in the cervical third should be routinely treated under the microscope via standard root canal therapy (RCT) approaches. The apical portion of the root canal system has been considered critical for therapeutic and pathogenic reasons [33], with cleaning isthmuses in this region being essential to effective long-term treatment outcomes. Even when appropriate irrigation and preparation techniques are used, preventing and controlling infections in this site can be challenging. We determined that 17.36% of cases in our study exhibited isthmuses in the apical third region, although limitations in the CBCT technology may mean that the true prevalence rate is even higher as the apical structure is relatively subtle. Fortunately, treatment of this region via apical surgery typically yields favorable clinical outcomes [34]. The treatment of the apical area by apical surgery often achieves ideal clinical results, although surgical success rates are highest in teeth that do not have an isthmus relative to those with a prepared isthmus [35]. Given these success rates and the fact that there is a risk of weakened roots following preparation [36], efforts must be made to improve isthmus preparation techniques for isthmuses located in the apical region. Enhanced efforts to understand how such isthmuses affect surgical outcomes will make it possible to ensure better patient results [2]. Isthmuses located in the middle third region are relatively more difficult to effectively clean in the process of apical surgery or RCT. Substantial amounts of tissue may be destroyed to access isthmuses in this location, potentially significantly increasing fracture rates. We found that the prevalence rate of such isthmuses was 18.48%, which is fairly low. While it is important to clean isthmuses located in the middle third section, they are not as prevalent as those in cervical or apical regions. Given the risks of operating in this site, conservative measures should be taken during treatment.

Given the relatively strict definition which we used, we detected a very low prevalence of true MM canals by CBCT. Our and others’ results suggest that true MM canals should only be identified when three distinct independent canals are present. Detecting and managing isthmuses presents as an important but complex challenge. Navigating both isthmuses and MM canals can be accomplished using a combination of imaging approaches, tactile search techniques, and an operative microscope to carefully and accurately navigate these delicate and complex structures. Future studies assessing long-term patient outcomes are needed to better explore the effects of isthmus and MM canal preparation on non-surgical endodontic treatments in mandibular first and second molars.

## Conclusions

CBCT could be a useful tool for detecting MM and isthmus, we found high prevalence of isthmuses and low prevalence of MM in mesial root of mandibular first molar in a Chinese population. Moreover, mandibular first molars with a separate distal root or from the elderly tend to have a lower isthmus prevalence. As a leading cause of the failure of endodontic treatments for mandibular molars is the presence of a canal isthmus [14], detecting and cleaning these areas during root canal therapy and apical surgery is a critical step.

## Supplementary information

**Additional file 1.** Raw data.

## Data Availability

The original data analyzed during the present study are available in supplementary material (raw data).

## Abbreviations

CBCT: Cone Beam Computed Tomography
MM: Middle Mesial
M: Mesial
D: Distal
L: Lingual
B: Buccal
mm: millimeter
RCT: Root Canal Therapy
